# Study of enzymatic detergents with both cleaning efficacy and disinfecting action for medical devices

**DOI:** 10.1186/2047-2994-4-S1-P37

**Published:** 2015-06-16

**Authors:** Y Kato, Y Toga, Y Oda, E Kawamukai, Y Hirata

**Affiliations:** 1 Biochemical Laboratory, SARAYA CO., LTD., Osaka, Japan

## Introduction

Meticulous cleaning of used medical devices with alkaline or enzymatic detergents should remove organic debris, minerals, bioburden and other contaminants. Some enzymatic detergents not only have cleaning efficacy, but with additional disinfecting action as well. Having dual performance can reduce bioburden in the first step of medical device reprocessing, as well as and can prevent the discharge of contaminated wastewater to the environment. However, as disinfecting agents are found to denature and fix proteins on medical devices, making it difficult to perform both cleaning and disinfecting at the same time.

## Objectives

Enzymatic detergents with incorporated disinfecting agents were investigated for their cleaning efficacy and disinfecting action.

## Methods

The cleaning efficacy was verified with cleaning indicator ‘TOSI®’, simulating dried blood, which is commonly used to evaluate the cleaning efficacy on medical devices. After immersing TOSI® at 40 degrees Celsius for 30 minutes, it was visually evaluated according to the presence of residual blood. The disinfecting action was then verified with methods based on EN13727 and EN13624.

## Results

When disinfecting agents were incorporated to enzymatic detergents, it was found that the cleaning efficacy decreased with increasing of disinfecting action, as observed in other commercial enzymatic detergents having dual performance. To promote balance of both cleaning and disinfecting performance, approximately thirty surfactants were investigated, and among them, two were selected as suitable cleaning agents. Incorporating the optimum amount of disinfecting agents, it is then possible to achieve the desired dual performance.

## Conclusion

Generally, it is difficult to achieve both cleaning efficacy and disinfecting action in enzymatic detergents. Disinfecting agents are known to fix proteins, hindering the cleaning action of enzymatic detergents. On the other hand, increasing the amount of cleaning agents to improve the cleaning efficacy can then decrease the disinfecting efficacy. This study suggests that suitable balance of cleaning and disinfecting agents in the formulation will make it possible to achieve both the desired, effective cleaning and disinfecting performance in enzymatic detergents for medical device reprocessing.

## Disclosure of interest

None declared.

